# The Influence of Initial Beliefs on Judgments of Probability

**DOI:** 10.3389/fpsyg.2012.00381

**Published:** 2012-10-05

**Authors:** Erica C. Yu, David A. Lagnado

**Affiliations:** ^1^Cognitive, Perceptual and Brain Sciences, Division of Psychology and Language Sciences, University College LondonLondon, UK

**Keywords:** model-based learning, belief revision, priors, gambling, probability judgment

## Abstract

This study aims to investigate whether experimentally induced prior beliefs affect processing of evidence including the updating of beliefs under uncertainty about the unknown probabilities of outcomes and the structural, outcome-generating nature of the environment. Participants played a gambling task in the form of computer-simulated slot machines and were given information about the slot machines’ possible outcomes without their associated probabilities. One group was induced with a prior belief about the outcome space that matched the space of actual outcomes to be sampled; the other group was induced with a skewed prior belief that included the actual outcomes and also fictional higher outcomes. In reality, however, all participants sampled evidence from the same underlying outcome distribution, regardless of priors given. Before and during sampling, participants expressed their beliefs about the outcome distribution (values and probabilities). Evaluation of those subjective probability distributions suggests that all participants’ judgments converged toward the observed outcome distribution. However, despite observing no supporting evidence for fictional outcomes, a significant proportion of participants in the skewed priors condition expected them in the future. A probe of the participants’ understanding of the underlying outcome-generating processes indicated that participants’ judgments were based on the information given in the induced priors and consequently, a significant proportion of participants in the skewed condition believed the slot machines were not games of chance while participants in the control condition believed the machines generated outcomes at random. Beyond Bayesian or heuristic belief updating, priors not only contribute to belief revision but also affect one’s deeper understanding of the environment.

## Introduction

When a decision making process occurs over a temporally extended interval, new evidence may accumulate over time that warrants the updating of initial beliefs. Particularly in novel environments where initial beliefs may be based on only scant information, how should we integrate our initial beliefs about the environment with subsequent observations?

Combining a prior description with experience is an everyday activity, as mundane as judging the likelihood that it will rain later in the afternoon, given a weather forecast and one’s own observations from looking out the window. Consider, for example, slot machines. At most slot machines today, the only information known to players before the start of a game is a long-run payout percentage and a succinct payout table that lists which outcomes are associated with which combinations of symbols (or, as some might have you believe: which combinations of symbols *cause* which outcomes). This incomplete description of the environment lacks probability information for the listed outcomes. Players must repeatedly play the machines to learn about the missing probability distributions and learn which machines may be most valuable to play at. In other words, the slot machine is an inductive inference problem. Gambles are of particular interest to the study of inductive inference because they are so similar to everyday reasoning and yet very dissimilar at the same time. Due to the underlying randomness in many gambles (such as slot machines), people who fare well in everyday reasoning can fail in the face of gambles. Indeed, it is still a mystery why people can be so good at some gambles (such as poker) and yet so bad at others (roulette). How can we account for this?

In this paper, we investigate the impact of initial descriptive information about an environment of uncertainty on judgments about the environment’s structure. Our study extends previous work on the topic by investigating the impact of induced priors as well as evidence on judgments and beliefs about structure. To accompany the slot machines in our full-feedback paradigm, we provide payout tables, thereby controlling the initial beliefs of which outcomes are possible. However, we do not provide the probability distribution nor set one machine to be more profitable. Instead, we manipulate whether the evidence is congruent or incongruent with prior beliefs. How will participants update their prior beliefs given this evidence? To anticipate our results, we find that neither simple Bayesian nor heuristic-based accounts of belief revision can explain our findings; instead, a model-based framework is proposed for a descriptive account of decision making over time.

Prior knowledge is a central factor of decision making theories, across the spectrum from heuristic (gambler’s fallacy: a prior regarding representativeness of outcomes; Tversky and Kahneman, 1971; Kahneman and Tversky, 1972; anchoring and adjustment: insufficiently adjusting estimates from a reference point; Tversky and Kahneman, 1974) to rational (game theory: consistency of prior beliefs between players in a game; Harsanyi, 1967). Likewise, research has demonstrated that different assumptions for priors can lead to different posterior beliefs (Troutman and Shanteau, 1977; Koehler, 1993) as can prior outcomes affect subsequent judgments and decisions (Thaler and Johnson, 1990). Decision makers may maintain their initial hypotheses by dismissing disconfirming evidence (Klayman and Ha, 1987) or even inappropriately using disconfirming evidence to support initial hypotheses (Snyder and Swann, 1978; Doherty et al., 1979; Fischhoff and Beyth-Marom, 1983). Under uncertainty, prior beliefs can have a cascading effect on subsequent judgment and decision making that is deeper than mere belief-adjustment.

The model-based approach to understanding learning uniquely captures this relationship between prior and posterior beliefs. It derives psychological validity from theories of internal representations of external events or ideas. Well-known examples of internal representations in the literature include schemas, structures of knowledge that include concepts of components, attributes, and relationships between specific instances (Simon, 1957; Schank and Abelson, 1977; Bower, 1981; Pearson et al., 1984), and mental models, internal symbolic understanding of the external world (Johnson-Laird, 1983). Models in this sense are small-scale representations of reality, neglecting facts, and relationships that are outside one’s scope of knowledge with cascading effects on reasoning. As in previous theories of decision making that use models, this approach includes representations of the states of the environment, actions, and rewards (as opposed to model-free learning, which assumes no representation of structure; Dayan and Niv, 2008); however, the present model-based approach focuses on *inference* in contrast to optimized decision making. A close and very interesting example of model-based inference can be found in Lopes (1976) though here, too, the emphasis is on how to optimize betting rather than how models influence understanding of the structure of the game. Although computational model-based learning considers the value of these representations in the construction of a model of the environment, the present perspective emphasizes representations of explanatory, causal, and goal-directed beliefs about the relationships between these components.

**Figure 1:**
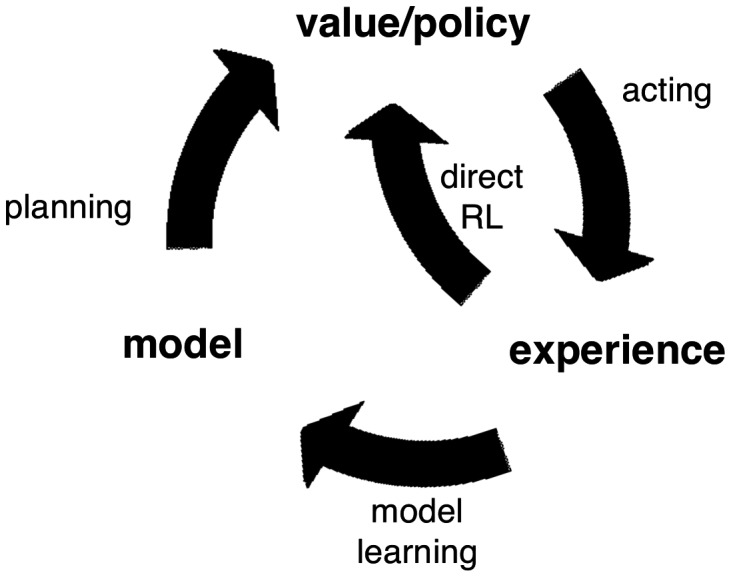
**The cycle of model revision over time (Sutton and Barto, 1998, p. 231)**.

The top-down structure enables the agent to reason broadly and make inferences about classes and categories of events and relationships using prior knowledge as well as in a more detailed manner about the specifications of the current problem using data. The revision process, in which models are updated as new evidence is acquired, utilizes the structure of the models to exploit previous experience and knowledge, including information about the relationship between events such as data-generating processes, not just the events themselves (Sutton and Barto, 1998). The learner evaluates new information against his or her prior beliefs, much like Edwards (1968) and the belief-adjustment model of Hogarth and Einhorn (1992) in which new evidence is added or averaged with previous information using anchoring and adjustment. However, within the model-based framework, new evidence can be observed without requiring adjustment to the model. The overall process can be depicted as a feedback loop whereby the outcomes of the learner’s actions are fed back into his or her beliefs about the problem. Figure 1 illustrates this loop: the consequence of an outcome after an agent’s action may be to update the policy directly or to feed back to the agent’s model and be integrated with prior beliefs. For example, fluctuations in outcome within a range of expected possible outcomes, such as those in scenarios with random processes, need not change the learner’s model of the environment (Yu and Dayan, 2005). A player at a slot machine may avoid a machine after experiencing losses or, depending on his model of the outcome-generating process, stay at the machine because a win is more likely to occur after a string of losses. A poker player may fold after observing another player raise or, depending on his model of the player, re-raise because he believes based on previous games with that player the raise to be a bluff. Because models of the environment are highly structured and include histories of previously held beliefs, new information can go beyond adjustments to cause qualitative and systemic changes in models.

Critically, as a consequence of these two dissociable components of model and belief revision, an individual may appear to behave irrationally while implementing rational inference. If the player at the slot machine holds a model of the game whereby a string of losses is certainly followed by a win, then he acts rationally when he continues to play despite losing. If the model of the problem is inaccurate, it may be the case that new evidence does not correct errors despite the correct implementation of the updating process. The individual may persist in believing inaccurate information.

The model-based learning framework is not commonly cited in psychological research on decision making. Researchers more often appeal to rational Bayesian accounts (Edwards et al., 1963; Steyvers et al., 2003; Tenenbaum et al., 2006). Bayes’ theorem, a general mathematical rule commonly used for belief updating using evidence, results in a posterior probability expressing the degree of belief about the likelihood of a hypothesis being true after observing data. However, while Bayesian inference itself may be straightforward, the assumptions made about the hypothesis sets and approximation algorithms are less clear, and sometimes lacking in theoretical and empirical grounding (Jones and Love, 2011). The psychological implications as regards to the cognitive capacity required to consider all evidence even-handedly, generate exhaustive sets of hypotheses, and calculate likelihoods are out of reach for most people, including experts (Meehl, 1954; Fischhoff et al., 1978; Fischhoff and Beyth-Marom, 1983).

To study the integration of prior beliefs with evidence accumulated over time, we use a slot machine modification of the “computerized money machine” typically used in decision-from-experience tasks (e.g., Barron and Erev, 2003). Two slot machines each present a “button” to push that offer probabilistic payouts based on distributions unknown to the participant at the start. One difference to previous experiments is that, in the present task, the machines are identical. As we are primarily interested in how the descriptive information is combined with experience, we did not complicate the task further. A second critical difference is that the slot machine provides additional descriptive information to the participant in the form of a payout table that lists the space of potential outcomes. This will be discussed in greater detail below.

Previous work using this decision making over time paradigm has focused on finding differences between problems based on description and those based on experience (Barron and Erev, 2003; Hertwig and Erev, 2009; Ungemach et al., 2009). This paper focuses instead on an area slightly outside of that dialog: the combined effect of description and experience. Newell and Rakow (2007) discuss this research question with a binary prediction game using dice for which participants are given outcome probabilities and the opportunity to experience outcomes over time. When given only abstract, descriptive information about the outcomes and probabilities, participants were likely to choose the optimal strategy; when given both abstract information and experience, participants were more likely to sub-optimally probability match. Although this finding reveals the combined effect of descriptive and experiential information on risky choice, it does not provide a deeper insight into the cognitive processes participants may be using in such tasks. Strategy probes questioned participants only at the end of games and did not capture the reasons behind response choices. Critically, with dice stimuli that represent a definitively random outcome-generating process with known probabilities, questions of how participants learn about structure and probability distributions under uncertainty are outside the scope of that study.

To test the effects of prior knowledge on learning, we manipulate the content of the prior. Some participants were induced with the “control” condition prior, which accurately reflected the abstract outcome information for the task to be played: the slot machine payout table included all valid outcomes only. In contrast, other participants were induced with a skewed prior, which reflected the same information as controls with the addition of fictional higher outcomes. All participants played the same machines and observed effectively the same evidence. What effect would the different priors have on beliefs about structure and probability as new evidence was attained?

## Materials and Methods

### Participants

Fifty-three participants were recruited to participate in paid studies on gambling from the University College London Psychology Department Subject Pool, a popular online UK notice board, and local newspapers. All individuals gave their informed consent to participate in the experiment, as approved by the Department of Psychology at University College London.

### Design

The design comprised one within-subjects factor over time with three levels (before the start of the game, after 30 trials, after 80 trials) and one between-subjects factor with two levels (congruency of payout table and outcomes, “skewed” in congruency). Participants were randomly assigned to conditions, resulting in a final sample of 27 participants in the congruent payout table group and 26 participants in the skewed payout table group. Participants played 80 mandatory pulls on the machines and answered questions before the start of play, after 30 arm pulls, and at the end after 80 arm pulls; machine choice and pace were up to participants but the total number of pulls played was fixed.

All participants played the same task with equivalent slot machines that sampled from the same underlying distribution of outcomes: 0, 2, 3, 4, 5, and 10. Participants in the control condition were shown a payout table before the start of the game that displayed the outcome values of the game and their associated reel symbol combinations (i.e., congruent with the observed evidence: 0, 2, 3, 4, 5, and 10). The other group of participants was shown the same payout table, but now with fictional higher-value outcomes (i.e., incongruent with the observed evidence: 0, 2, 3, 4, 5, 10, 15, 20, 25, and 100). Despite the different payout tables, both groups played the same task and observed random draws from the same underlying distribution. No participants were shown the probabilities associated with the outcomes. The skewed table suggested an initial belief that overlapped with the control participants’ but also included fictional higher-value outcomes that would not be observed during the experiment^1^.

Participants were randomly assigned to either the experimental skewed or the control condition and remained unaware of any alternative task specifications. In each condition, participants were informed that their compensation would depend on the bank’s value at the end of the task.

### Materials

The slot machines required a five pence stake for each play, which was taken from the £3.00 bank endowed by the experimenter to the participant at the start of the task. The machines used a random process to select outcomes from a fixed distribution: an outcome of 0, 2, 3, 4, or 5 pence with 17.4% probability each or 10 pence with 13.0%. The expected value of a play at any machine was 3.9 pence, at a loss of 1.1 pence given the cost to play.

The experiment interface used fruit graphics, which are highly associated with slot machines imagery, and animations such as spinning reels and moving levers to simulate the appearance of real-world slot machines. The size of the screen display allowed the participant to see up to three symbols on each reel depending on the reel position. The machines had a single payout line (combinations of symbols must fall on the payout line to qualify for winnings) through the middle; symbols above and below (near misses) were also viewable but randomized. When a participant clicked on a machine to play it, the reels appeared to spin, and then slow to a rest after 3 s, displaying the final screen of fruit symbol graphics that matched the outcome received on that trial. The final screen and numerical outcome value (e.g., four pence) was shown on the screen for 1.5 s. The screen then returned to the initial state and participants could click on the machine they wished to play next. No bank or cumulative total information was shown to the participant at any time during the task except after completion.

When asked to illustrate their beliefs about what outcomes the machines paid out and how likely those outcomes were, participants were given blank pie chart templates and pens. Each template sheet had one blank circle for the machine on the left and another identical one for the machine on the right, each with an indicator for the center of circle to aid in drawing. Most participants readily understood this instruction but all watched the experimenter create an example pie chart and had the opportunity to ask questions. This method was chosen both for its familiarity for participants and for its convenience for eliciting comparable judgments between groups of the size of the outcome space. By providing blank response templates, this design requires participants to generate the outcome space and associated probabilities without being prompted by the experimenter for different outcome values. Alternative methods of elicitation of probabilities, such as prompting responses one outcome at a time, or listing the full space of outcome values for the participant, may have defined the outcome space for the participant and consequently rendered their subjective probability distributions invalid. Although phenomena such as sub additivity cannot be investigated in this paradigm, the benefits for the relevant hypotheses being tested are greater than this limitation.

### Procedure

At the start of the task and at two times during the task, participants were asked to answer questions. Before beginning machine play but after being shown the playing environment including the machines and payout tables, participants were asked to give their best guess as to the hidden probabilities associated with the outcome distribution. After completing a pie chart for the machine on the left of the screen and another for the machine on the right, participants then began to play the machines. After 30 trials, the program automatically stopped and prompted participants to respond to questions. The experimenter presented pen and clean paper and asked the participant to again illustrate the different payouts they believed the machines generally paid out, and how likely those payouts were. After 80 trials, the prompts were repeated. The timing of the prompts after 30 and 80 trials was not known to the participants. The payout tables that displayed the outcome space were visible during the first response time before play had begun but were not visible during the latter two judgment collections; participants completed these pie charts from memory.

After completing the 80 trials, participants responded to a forced choice question about the machines’ outcome-generating processes. Participants were asked which statement most closely matched their belief: “playing required skill to avoid bad luck or bad streaks at machines” or “it did not matter what I did or how I played.”

## Results

In this experiment, a mixed design compared two groups’ changing beliefs over time about a hidden outcome distribution, measured by probability judgments and responses to direct questions after completion of the task. Neither group was compared to the true underlying distribution because an infinite number of processes might have produced the sequences observed by the participants; only summary statistics (expected value) of the judged probability distribution were compared to the observed distribution. The experience of the two groups varied only on the range of outcomes listed in the payout tables shown during the task; all participants experienced slightly different sequences of outcomes due to random sampling but no significant differences in the final sum of outcomes received were found between groups, *t*(52) = 1.23, ns.

### Estimates of mean payout

**Figure 2:**
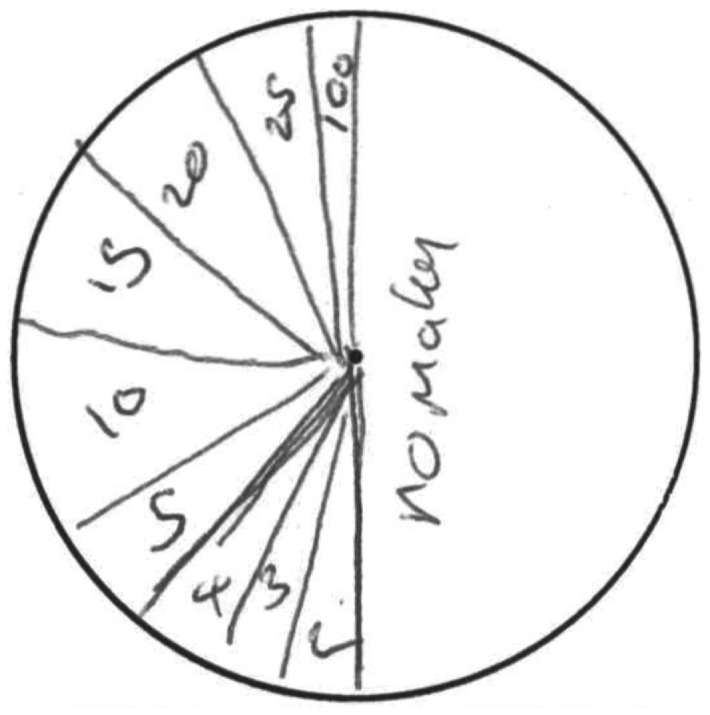
**Example of a participant’s hand-drawn initial judgment of a machine’s underlying probability distribution of outcomes**. This participant indicated that the possible outcome space included 0, 2, 3, 4, 5, 10, 15, 20, 25, and 100 pence outcomes and that the most likely outcome would be of no matching symbols, or 0 pence.

Participants in both experimental groups made probability distribution judgments before play began, after 30 trials, and after 80 trials. At these collection times, participants were asked to report what payouts they thought the machine paid out in general, and how likely were those payouts. An example of a participant’s response in pie chart form is shown in Figure 2.

**Figure 3:**
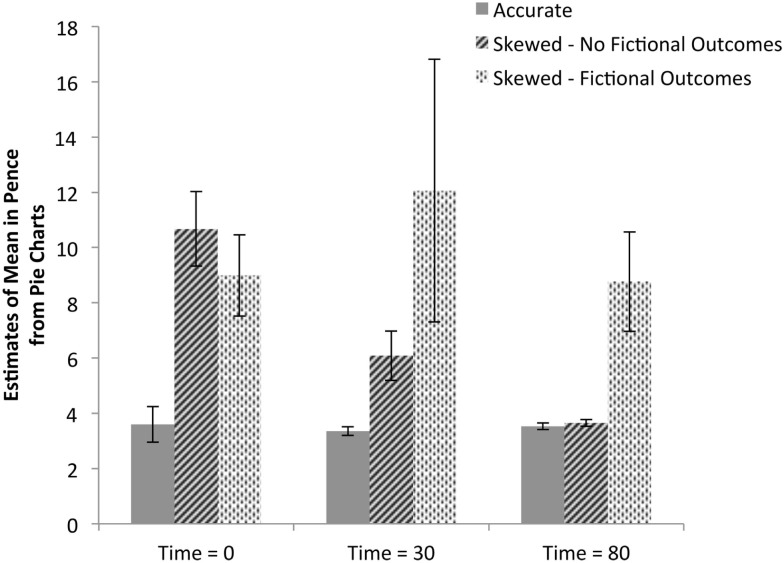
**Implied estimates of mean payout across time**. Data from participants’ pie charts were used to calculate each participants implied estimate of mean payout. Data shown in solid gray bars are from participants who were shown accurate payout tables including only valid outcomes. The data are split based on whether the participant persisted throughout the game in including the fictional higher-value outcomes of 15, 20, 25, and 100 pence; 8 of 26 participants given skewed payout tables included the fictional outcomes; no participants given accurate payout tables did so. Responses for the left and right machines at each judgment collection time were averaged resulting in one response per participant for each of the three judgment times.

The first analysis of these data is of the raw mean estimates calculated using participants’ pie charts, as shown in Figure 3. By measuring each pie chart segment, we were able to assess how likely the participant believed each outcome to be and therefore the participant’s subjective expected value for a play of the slot machine. In the example shown in Figure 2, the participant expresses a belief that the probability of an outcome of 0 pence is 50% and the subjective expected value of a play of the machine is 7.74 pence. Estimates of the left and right machines were averaged (no differences between expected value estimates given for left and right machines (paired samples *t* tests, all *p*s > 0.24), resulting in a single data point for each subject at each time point. Analysis of these data finds that there are significant differences between the two experimental groups [*F*(1,49) = 29.95, p < 0.001, $\eta_{\text{p}}^{2}=0.38$], demonstrating that, overall, the payout table with fictional higher-value outcomes led to higher valuations of the slot machines. Significant effects were also found within groups over the three judgment times [*F*(2,98) = 4.18, p < 0.02, $\eta_{\text{p}}^{2}=0.08$]; *post hoc* Bonferroni-adjusted comparisons show the difference lies between the first and third judgment times only (*p *< 0.01; between first and second: *p *< 0.92 and between second and third: *p *< 0.09). In other words, valuations gradually decreased. The payout table and time interaction was also significant [*F*(2,102) = 4.12, *p *< 0.02, $\eta_{\text{p}}^{2}=0.08$] with follow-up tests showing that the payout tables with the fictional higher-value outcomes led to higher valuations compared to the control group at all judgment times (all *p*s < 0.02). These findings suggest that participants who were shown skewed payout tables consistently gave higher estimates of the expected value of a machine play than control participants, but showed a trend of converging toward observed values as a function of number of trials played. Splitting those participants who were shown skewed payout tables further into those who persisted in representing fictional higher-value outcomes throughout the duration of the game (shown in Figure 3) suggests that this difference is primarily driven by the representation of higher-value outcomes rather than misestimates of the probability of the true observed outcomes.

**Figure 4:**
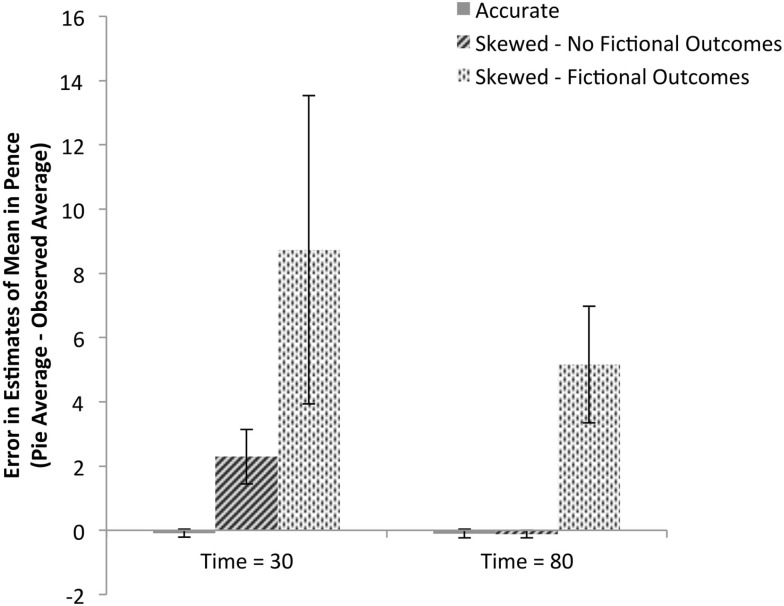
**Mean error in payout estimation based on condition and belief in fictional outcomes**. Each participant’s mean observed payout for each machine was subtracted from each participant’s pie chart estimate of mean payout for that machine to calculate errors in estimation. The data are split based on whether the participant persisted throughout the game in including the fictional higher-value outcomes of 15, 20, 25, and 100 pence; 8 of 26 participants given skewed payout tables included the fictional outcomes; no participants given accurate payout tables did so. Values greater than zero indicate overestimation and values close to zero indicate accurate estimation. Errors for both the left and right machines were averaged resulting in one error measure per participant per judgment during machine play.

Remembering that each participant observed a different randomly generated sequence of outcomes, the next analysis illustrated in Figure 4 examines each participant’s pie estimates of the expected value given their unique observed sequence of outcomes to assess whether group differences in estimates are due to differences in observed sequences. Their observed values were compared to their reported estimates from collection times after 30 and 80 trials, with the left and right machine estimates averaged for each participant, to calculate an error measure; initial judgments are not included in this analysis because participants did not observe any outcomes before making initial judgments. These data confirm the analysis of the raw estimates: there are significant differences between the two experimental groups [*F*(1,51) = 7.06, *p *< 0.01, $\eta_{\text{p}}^{2}=0.12$] and within groups over the three judgment times [*F*(1,51) = 6.45, *p *< 0.01, $\eta_{\text{p}}^{2}=0.11$]. The interaction of the two factors is also significant [*F*(1, 51) = 6.29, *p *< 0.02, $\eta_{\text{p}}^{2}=0.11$].

### Accuracy of pie estimates

As shown in Figure 4, control participants who were shown an accurate payout table made precise estimates not significantly different from the observed data after 80 trials [M = −0.09, SE = 0.13; one-sample *t*-test against 0: *t*(26) = 0.67, ns] and after only 30 trials [M = −0.10, SE = 0.13; one-sample *t*-test against 0: *t*(26) = 0.77, ns]. This accuracy provides support for the validity of this method of subjective probability elicitation to capture sensible data.

### Representation of fictional higher-value outcomes

Although it is evident that the participants of the two groups perceive the expected value of each machine play differently, further analysis of the pie charts may explain this difference. Estimates of the means alone cannot distinguish overestimation of the likelihood of observed outcomes (e.g., believing the 5 or 10 pence outcome happen more frequently than the observed data suggest) from categorically representing higher-value outcomes with any degree of likelihood (e.g., believing the 100 pence outcome is possible with 1% probability). The pie charts show that the primary source of the overestimation comes from maintaining a belief in the likelihood of the fictional higher-value outcomes. After 30 trials, 61.54% of participants (16 of 26 participants) in the skewed payout table group continued to maintain the unsupported belief of at least one higher-value outcome while only 3.70% (1 of 27 participants) indicated the same in the control group (*p* < 0.001, Fisher’s exact test). After even 80 trials, the difference in number of participants maintaining beliefs in higher-value outcomes remains significant (Skewed: 30.77% or 8 of 26 participants; Controls: 0%; *p* < 0.01, Fisher’s exact test). This pattern shows that participants converged toward the observed data and no participants developed a skewed belief of unsupported fictional higher-value outcomes after having expressed a belief reflecting the observed outcome values only.

### Beliefs about the nature of the outcome-generating process

**Figure 5:**
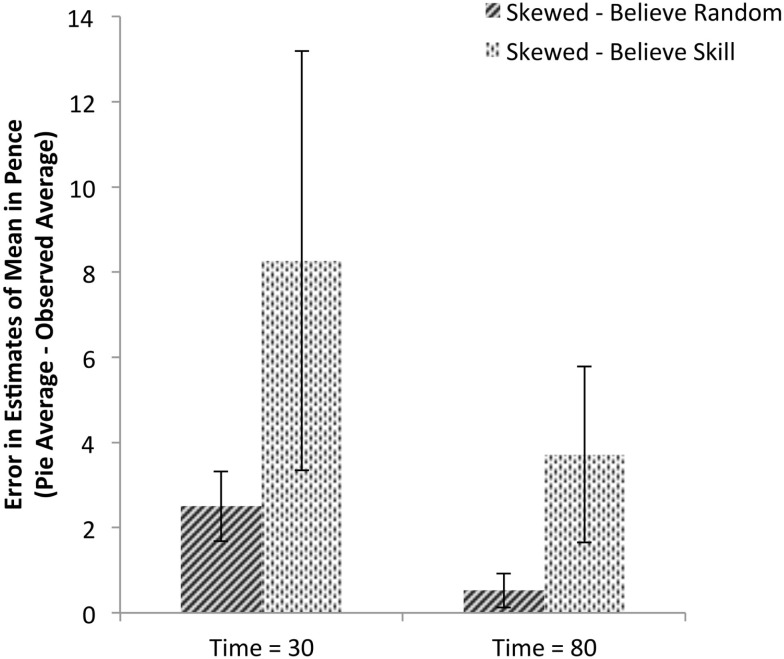
**Mean error in payout estimation based on structural beliefs**. Mean payout estimate errors are shown for those participants given skewed payout tables, split based on whether the participant responded with the belief that the machine play was random or based on skill (8 of 26 participants); data from the control group of participants shown accurate payout tables is not included because only 1 of 27 participants expressed belief that machine play was based on skill. Values greater than zero indicate overestimation and values close to zero indicate accurate estimation. Errors for both the left and right machines were averaged resulting in one error measure per participant per judgment during machine play.

A direct question probed participants for their beliefs about the nature of the underlying outcome-generating process. Although participants were probed only once after the completion of the task, this assessment enables us to infer, assuming no differences in beliefs about the outcome-generating processes of slot machines before beginning the task, whether the task environment changed participants’ beliefs. It was hypothesized that participants who were shown the skewed payout table and expected to receive higher-value outcomes may believe that they are performing poorly on the task when they do not receive the expected outcomes. When asked whether the outcomes were generated by a random process (a choice between the two statements: “playing required skill to avoid bad luck or bad streaks at machines” or “it did not matter what I did or how I played”), only 3.84% (1 of 26 participants) in the control group indicated that skill was a significant factor while 30.77% (8 of 26 participants) indicated this from the group who viewed skewed payout tables. This analysis suggests that the different payout tables influenced the participants’ beliefs about the outcome-generating process. This difference between groups within the Skewed condition is shown in Figure 5: those participants who believe skill to be involved in machine play estimate the machines to be significantly more valuable than the machines actually are. Indeed, the two are highly related: a logistic regression predicting belief type finds that those participants who categorically represent at least one fictional higher-value outcome are 8.00 times more likely to also believe the outcome-generating process is based on skill (B = 2.08, *p *< 0.02). Although these correlations cannot provide directional explanations for participants’ responses, they support the hypothesis that beliefs about an underlying outcome distribution and associated underlying outcome-generating process may be related.

## General Discussion

In this study, we induced different initial beliefs about the task environment and observed how the accumulation of evidence changed those beliefs. The initial beliefs informed participants of the outcome space in the environment; some participants were given information congruent with what they would subsequently observe while others were given incongruent information (the description of the outcome space included fictional higher-value outcomes). Participants who predicted that the machines would produce fictional higher-value outcomes did so despite never observing their occurrence, and ultimately attributed the absence of those outcomes to (lack of) skill. From this experiment, there are two broad findings: participants integrated observed evidence with their initial beliefs for probability judgments but relied heavily on initial information to understand the structure of the environment. In other words, prior beliefs are more than just initial information; priors are the basis of internal models that affect beliefs about the structure of the data-generating processes.

When participants created pie charts from blank templates, they had to express not only the probabilities they thought were associated with outcomes but also the outcomes themselves. Despite the limitations of this study due to possible error in manual measurement of participants’ responses, the pattern of categorical responses is clear. The pie charts showed that many participants who initially believed that higher-value outcomes would occur continued to believe so even after playing 30 and 80 trials and never seeing those outcomes occurring. Observed evidence alone cannot account for this. The results are contrary to most memory-based theories that would predict the non-observance of these outcomes resulting in their absence from the subjective probability distributions, including those based on availability (Tversky and Kahneman, 1973). Similarly, model-free theories, in which judgment is based solely on experience, are also unable to account for this. Alternatives such as Bayesian accounts are more successful in explaining the role of prior beliefs in these judgments. Bayesian belief revision corresponds with the convergence of judgments over time and the variable weight on prior beliefs relative to observed evidence. From this perspective, participants’ skewed judgment in persistently predicting the fictional higher-value outcomes despite their non-occurrence can be seen as a rational belief: not enough evidence had been presented to outweigh the initial belief in the higher-value outcomes.

Up to this point in the analysis, Bayesian accounts are consistent with the behavior we found in this task. However, basic Bayesian theories do not specify the underlying cognitive process and have no explanation for the differences in the beliefs of randomness in the outcome-generating process required to explain the critical results. The evident difference in beliefs about how the outcomes are generated dictates that more is needed to explain the overall results – beliefs about the *structure of the environment* is the critical element. The model-based approach, which relies on the player’s representation and understanding of the structure of the game, tolerates the updating of probabilities based on evidence in parallel with the persistent representation of higher-value outcomes on the basis of no observed evidence at all. Although we probed participants only once for their attitudes regarding the data-generating process, those responses considered along with the probability judgments illustrate that initial beliefs have qualitative effects on subsequent judgments of probability and understanding of the structure of the environment. Future research should aim to capture a richer understanding of belief revision and decision making strategies by using additional measures and analysis methods, such as measuring probability judgments more often or such methods as those used in Jansen et al. (2012). This experiment demonstrates that theories of decision making must account for beliefs about the underlying data-generating process. In this broad sense, both heuristic and Bayesian theories that either approximate or explicitly include such causal beliefs may adequately describe the behavior in this task (Krynski and Tenenbaum, 2007). Future research should seek to refine and enrich our understanding of model structure.

Gambles are effectively inference problems in which the player must learn about hidden underlying outcome distributions and outcome-generating processes by generalizing from samples. Slot machines are a paradigm in which decision makers must learn over time about the value of their actions. While a die has six sides each with equal likelihood of landing face-up, a slot machine has an unknown number of symbols in unknown locations and ratios on each reel and an unknown algorithm determining the outcomes. Ultimately, slot machines pose the highest risk among games of chance because the unknown probabilities permit players to persist in believing (Griffiths, 1990). A question often raised is, “How many times did the individual continue to bet despite losing?” But it is the nature of the data-generating process that informs the predictability of the game and the rationality of a wager. It is the player’s representation of the game and how outcomes are generated that determines whether he succeeds in learning or persists in failing in the face of uncertainty. The models internal to the player are critical to understanding why people can persist in gambling despite losses (Gilovich, 1983; Walker, 1992). The conclusions from this research could be applied to future treatment research, improving targeted efforts to modify beliefs about the data-generating processes underlying gambles. Cognitive-based treatments have shown therapeutic gains (Bujold et al., 1994; Ladouceur et al., 1998); however, our research suggests that further efficacy gains may be made by focusing on the patient’s internal model of the gamble rather than teaching general principles of randomness. Similarly, simple changes to game infrastructure, such as displaying the probabilities of all outcomes rather than only the long-run expected value, may reduce inappropriate beliefs about the games. Gambles can be such difficult inference problems precisely because the player has so little information about his environment but must use his model nonetheless. Under uncertainty, the focus of our research should be on the cognitive process as well as the outcomes.

## Conflict of Interest Statement

The authors declare that the research was conducted in the absence of any commercial or financial relationships that could be construed as a potential conflict of interest.

## Footnotes

^1^Although the higher-value outcomes were fictional in this instance, the distribution is not dissimilar from real-world slot machines for which the desirable high-value outcomes are generated at infinitesimally low probabilities.
